# Regulation of Cytoskeleton Organization by Sphingosine in a Mouse Cell Model of Progressive Ovarian Cancer

**DOI:** 10.3390/biom3030386

**Published:** 2013-07-16

**Authors:** Amy L. Creekmore, C. Lynn Heffron, Bradley P. Brayfield, Paul C. Roberts, Eva M. Schmelz

**Affiliations:** 1 Department of Human Nutrition, Foods and Exercise, Virginia Tech, Blacksburg, VA 24061, USA; E-Mails: acreekmo@iupui.edu (A.L.C.); bbrayfield@gmail.com (B.P.B.); 2 Department of Biomedical Science and Pathobiology, Virginia Tech, Blacksburg, VA 24061, USA; E-Mail: cheffron@vt.edu

**Keywords:** ovariancancer, cytoskeleton, sphingosine, focal adhesions, MLCK

## Abstract

Ovarian cancer is a multigenic disease and molecular events driving ovarian cancer progression are not well established. We have previously reported the dysregulation of the cytoskeleton during ovarian cancer progression in a syngeneic mouse cell model for progressive ovarian cancer. In the present studies, we investigated if the cytoskeleton organization is a potential target for chemopreventive treatment with the bioactive sphingolipid metabolite sphingosine. Long-term treatment with non-toxic concentrations of sphingosine but not other sphingolipid metabolites led to a partial reversal of a cytoskeleton architecture commonly associated with aggressive cancer phenotypes towards an organization reminiscent of non-malignant cell phenotypes. This was evident by increased F-actin polymerization and organization, a reduced focal adhesion kinase expression, increased α-actinin and vinculin levels which together led to the assembly of more mature focal adhesions. Downstream focal adhesion signaling, the suppression of myosin light chain kinase expression and hypophosphorylation of its targets were observed after treatment with sphingosine. These results suggest that sphingosine modulate the assembly of actin stress fibers via regulation of focal adhesions and myosin light chain kinase. The impact of these events on suppression of ovarian cancer by exogenous sphingosine and their potential as molecular markers for treatment efficacy warrants further investigation.

## 1. Introduction

Ovarian cancer is the fifth leading cause of cancer deaths among women with a five-year survival rate of less than 30% [1]. Early diagnosis of the disease still confined to the ovaries significantly improves the survival of women (up to 94%) [2]. Therefore, the ability of ovarian cancer cells to exfoliate from the original tumor, disseminate throughout the peritoneal cavity, adhere to and invade at secondary sites determines the lethality of the cancer. The role of the cytoskeleton in cell motility, adhesion and metastasis has been well established [3]. The cytoskeleton provides structural support for the cell but also serves as a physical link that connects the extracellular physical, physiological and biochemical environment and the cell surface to intracellular signaling pathways and nuclear events that regulate proliferation, contact inhibition, anchorage-independent growth, and apoptosis [4,5]. During cancer development, changes in the cellular architecture enhance the cells’ plasticity, modulate cell-cell interactions and their dynamic adhesion, allowing for increased motility and invasion [4,6]. Changes in the cellular architecture are a requirement for the epithelial-mesenchymal transition and are evident by altered cell morphology, adhesion, and migratory capacity. They are often associated with altered levels of proteins such as E-cadherin, cytokeratin, vimentin, and increases in migration, invasion, and resistance to anoikis [7], indicating that the cytoskeleton is a key player in malignant progression and in metastasis.

Cytoskeleton changes were also evident during the malignant progression in the Mouse Ovarian Surface Epithelium (MOSE) cell system. The syngeneic MOSE cells, derived from C57BL/6 mice, have undergone spontaneous immortalization and transformation in cell culture [8]. MOSE cells go through discrete stages as they are cultured, and cell lines representing a premalignant, non-tumorigenic phenotype (MOSE-E), a transitional intermediate phenotype (MOSE-I), and a highly aggressive malignant phenotype (MOSE-L) have been established for both *in vitro* and *in vivo* applications. A microarray analysis has identified genes and their functional categories that may be associated with MOSE progression [9]. Among other categories such as metabolism [10], many up- or downregulated genes are coding for cytoskeleton subunits and their regulatory proteins. These changes are also observed in the human disease, indicating that the MOSE model recapitulates important events in human ovarian cancer and is a unique tool to investigate early and late events in ovarian cancer.

Parallel to the changes in gene expression levels, the microfilament, microtubule, and intermediate filament structures became increasingly disorganized during MOSE progression [9] which was accompanied by morphologic changes including decreased cell size, loss of contact inhibition during cell growth, and gain of the ability to grow in multiple layers, form spheroids *in vitro* and tumors *in vivo* [8]. These changes were associated with altered biomechanical properties. As determined by atomic force microscopy, both the MOSE cells elasticity and viscoelasticity decreases during neoplastic progression, rendering the cells less stiff and more deformable [11]; this is dependent on the actin cytoskeleton but not on the tubulin network [12]. Changes in the cellular architecture also affect the electrical properties of the MOSE cells, increasing the membrane capacitance of the more aggressive MOSE-L cells [13,14]. The biological ramifications of these events are not completely clear but one could envision that increased deformability would be an advantage for metastasizing cancer cells and identifies the cytoskeleton and its organization as a target for intervention strategies.

Sphingolipids have received much attention because of their involvement in the regulation of cell growth, death, motility, but also as perpetuators, mediators or modulators of aging, inflammation, cancer, and diabetes, to name a few. Sphingolipids are composed of a long chain sphingoid base backbone, an amide-bound fatty acid forming ceramide (Cer), and a diverse head-group on the 1-hydroxy group in complex sphingolipids. They are ubiquitously expressed in all eukaryotic cells, localized in cell membranes where they can have important structural functions. However, sphingolipid metabolites also function as lipid second messengers, generated by cells in response to growth factors, cytokines, cellular stresses and other factors. The activation of enzymes involved in sphingolipid metabolism allows for the fast bidirectional conversion of metabolites, each with distinct targets and functions. For example, Cer and sphingosine (Sph) are generally growth inhibitory and cytotoxic, while their phosphorylated counterparts ceramide-1-phosphate and sphingosine-1-phosphate (S1P) often mediate growth and survival, and are involved in inflammatory responses [15,16]. Therefore, the sphingolipid profile generated via modulation of enzymes in the sphingolipid metabolism is critically involved in the cellular response to intra- and extracellular stimuli. Cancer cells often exhibit aberrant profiles of sphingolipid metabolites that may promote tumorigenesis and metastasis; thus, the enzymes of sphingolipid metabolism have been identified as targets for chemotherapeutic interventions. In a different approach, exogenous sphingolipids have been used as anti-cancer agents, inducing cell cycle arrest or apoptosis in cancer cells, suppressing motility, invasion and angiogenesis [17].

Our studies have shown that dietary complex sphingolipids can suppress early and late stages of colon cancer in mice [18,19,20,21,22,23]. The regulation of proliferation rather than the induction of apoptosis was evident in all studies, which likely accounts for the lack of deleterious side effects noted using this route of administration. Throughout the intestinal tract, complex sphingolipids are hydrolyzed to the bioactive metabolites Cer and Sph, which are taken up by the intestinal cells; a small percentage of mostly free sphingoid bases reaches systemic distribution [24,25] in sufficient amounts to suppress experimental liver [26] and breast cancer [27] in rodents. Our recent studies have revealed a significant disruption of the actin cytoskeleton during malignant progression, suggesting a link between cytoskeleton dysregulation and neoplastic progression. Hence, in the present studies we hypothesized that the anti-cancer effects of Sph may be linked to modulation of the cellular architecture of tumor cells. Specifically, we investigated whether the cytoskeleton and its regulation are a potential target of bioactive sphingolipid metabolites that could be exploited to suppress ovarian cancer growth and metastasis.

## 2. Results and Discussion

### 2.1. Sph Treatment Increased Cytoskeleton Organization

**Figure 1:**
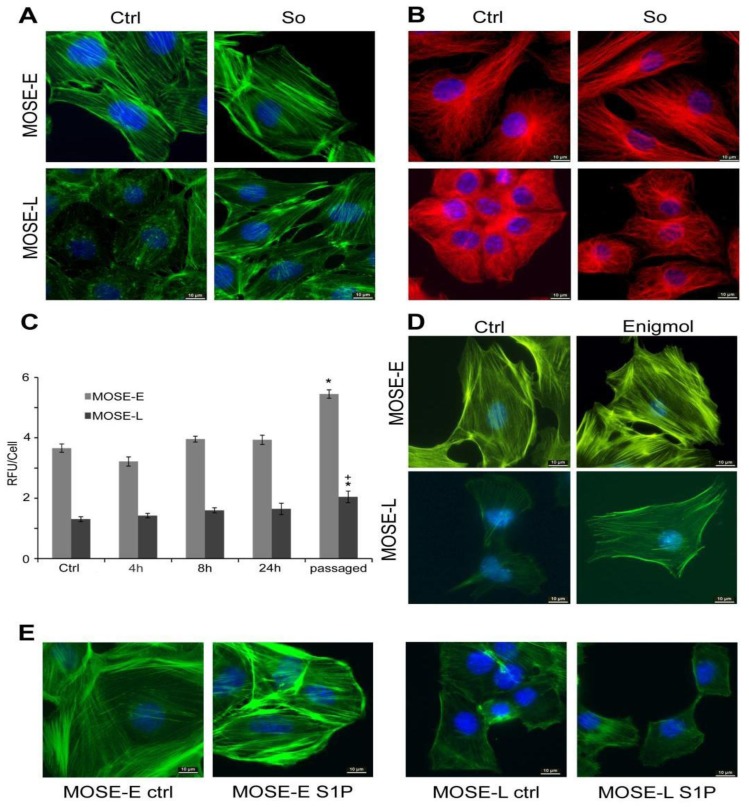
Sph treatment increases cytoskeleton organization. Mouse Ovarian Surface Epithelium (MOSE)-E and MOSE–L cells were grown in the absence (Ctrl) or presence of 1.5 µM sphingosine (So), fixed with paraformaldehyde and stained with AlexaFluor^488^-conjugated phalloidin to visualize filamentous actin (**A**) or with antibodies against ß-tubulin; (**B**) to visualize the microtubule network; (**C**) Quantitation of F-actin after 4–24 h treatment with 1.5 µM So, or continuous passaging. *significantly different from MOSE-E, *p <* 0.05, + different from untreated corresponding control, * p <* 0.05; (**D**) MOSE-E and L cells were treated with 1.0 µM enigmol for 3 passages and stained with AlexaFluor^488^-conjugated phalloidin; (**E**) MOSE-E and –L cells were treated with 500nM S1P for 8 h and stained with AlexaFluor^488^-conjugated phalloidin; (Original magnification X600).

To examine the influence of Sph on the progressively dysregulated cytoskeleton organization during MOSE progression, premalignant MOSE-E and malignant MOSE-L cells were grown in the absence or presence of 1.5 µM Sph for three to five passages. Importantly, this concentration of Sph did not result in overt toxicity to either MOSE-E or -L cells at any time point. As shown in Figure 1A, phalloidin staining of MOSE-E cells revealed a well-organized actin cytoskeleton architecture with prominent stress fiber formation that was enhanced upon Sph treatment. In contrast, untreated MOSE-L cells exhibited marginal stress fiber formation and F-actin cables were thin and highly unorganized as previously observed [9]. After Sph treatment, MOSE-L cells appeared somewhat larger and flatter, more similar to the MOSE-E cells; notably, the actin cytoskeleton became more organized, resulting in an increase in both the number and thickness of the actin stress fibers (Figure 1A, lower panels).

Sph can be metabolized by sphingosine kinase 1 and 2 (SK1, 2) to S1P; while there is only little information on S1P generated by SK2 [28], S1P generated by SK1 has been shown to influence the actin cytoskeleton organization in endothelial, muscle, immune, neuronal, and tumor cells [29]. Alternatively, Sph can be acylated to Cer that also can affect the actin cytoskeleton [30,31]. To address the question if the observed effects on the cytoskeleton are mediated by Sph or other metabolites, we treated cells with Cer and S1P; in addition, we also treated cells with DHCer, a metabolite considered biologically less active and often used as negative control but which has shown to induce autophagy [32]. The treatment with these sphingolipid metabolites did not affect the tubulin organization at any stage (data not shown). However, Cer and DHCer-treated MOSE-E cells exhibited somewhat thinner and shorter actin cables with more pronounced peripheral cables while there was little effect of S1P. Of note, S1P-treated MOSE-E cells began to lose contact inhibition of growth (supplemental Figure 1, upper panels). In MOSE-I, Cer, DHCer and S1P-treated cells exhibited more peripheral actin (middle panels, supplemental Figure 1). Neither treatment induced actin stress fiber formation in MOSE-L but both DHCer and S1P treated cells exhibited more microvilli-like protrusions on the cell surface (supplemental Figure 1, lower panel). This effect was more pronounced in MOSE-L than in MOSE-I. Since neither metabolite caused cytoskeleton re-organization that resembled the more benign cells, our following experiments focused on effects of Sph.

Sph treatment did not detectably alter microtubule organization in MOSE-E cells, which displayed long, defined, and well-organized microtubules (visualized by immunostaining for ß-tubulin) radiating out from the centriole (Figure 1B). In contrast, MOSE-L cells treated with Sph demonstrated less microtubule branching, a more defined centriole compared to untreated MOSE-L cells (Figure 1B, lower panels). The same effects were seen when the cells were stained for α-tubulin (data not shown). Sph did not have noticeable effects on the disorganized cytokeratin architecture in MOSE-L cells; the short, unorganized intermediate filament structures in MOSE-L [9] were not reverted to the well-organized network extending throughout the MOSE-E cells (data not shown).

### 2.2. Sph Treatment Increased Actin Polymerization Levels

To quantitate the Sph-induced changes in the microfilament network organization, a phalloidin-binding assay was employed to determine the effects of Sph on the amount of polymerized F-actin (Figure 1C). Untreated MOSE-E cells contained significantly more F-actin per cell than MOSE-L cells (over 2 fold higher, * p <* 0.05) (Figure 1C, ctrl), confirming our previous observations [9]. Sph treatment led to a gradual increase in the amount of polymerized f-actin in both MOSE-E and –L cells, reaching statistical significance (p < 0.05) only in cells that had undergone multiple passaging in the presence of Sph (*p ≤* 0.05). This suggests that prolonged Sph treatment is required either to maintain or stabilize the observed changes to f-actin organization. An increase in F-actin was also observed after treatment with enigmol (1.82 fold over non-treated control, * p <* 0.01) while treatment with Cer, DHCer or S1P did not show any significant changes (*p >* 0.05).

To confirm the effect of Sph, we treated MOSE cells with non-toxic concentrations of enigmol (2S,3S,5S)-2-amino-3,5-dihydroxyoctadecane) [33], a synthetic Sph analogue that lacks the 1-hydroxyl group and therefore cannot be phosphorylated by sphingosine kinases or utilized for the synthesis of complex sphingolipids. Comparable to Sph treatment, MOSE cells treated with 1 µM enigmol for three passages also displayed more stress fibers, an increase in actin organization and more spreading (Figure 1D), corresponding to the increase in F-actin increase (see above). Since S1P effects have been observed after brief incubation [34], we tested if S1P effect is detectable after shorter incubation. After 8 h of treatment with 150 nM S1P, the cells appeared somewhat flatter but only a sub-population of cells showed evidence of an increased actin bundling activity (Figure 1E). Taken together, these data suggest that Sph itself is at least partially responsible for the observed effects on increased actin organization and the conversion of Sph to S1P may not be required for the actin bundling activity.

### 2.3. So Increased the Expression of Actin Regulatory Proteins

**Figure 2:**
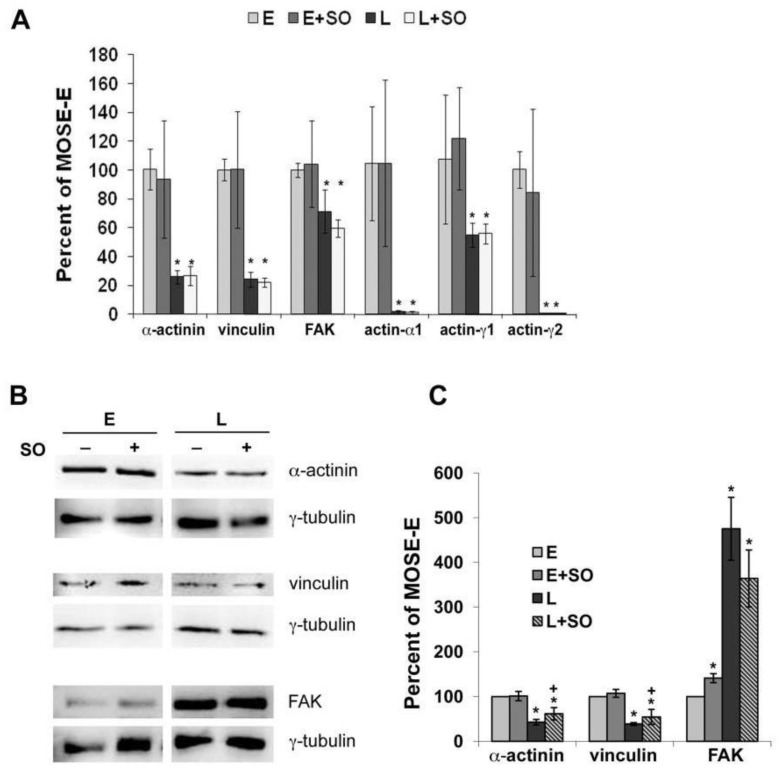
Sph treatment affects protein but not mRNA levels of cytoskeleton genes and regulators in MOSE cells. MOSE-E and MOSE–L cells were passaged three times in the absence or presence of 1.5 µM So. (**A**) Real-time PCR analyses of changes in mRNA levels of select cytoskeleton genes. *significantly different from MOSE-E, *p <* 0.05; (**B**) Representative Western blot of MOSE cells treated with So; and (**C**) quantitated using γ-tubulin as as housekeeping protein; expressed as percent of MOSE-E levels ± SD. *significantly different from untreated MOSE-E, p < 0.05; + significantly different from corresponding untreated controls, p < 0.05.

Previously, we reported altered mRNA and protein levels of α-actinin and vinculin, which together with FAK displayed altered subcellular localization patterns during MOSE progression. Since these proteins are involved in the organization of the actin cytoskeleton, we investigated if prolonged Sph treatment increases actin polymerization via modulation of these actin-regulating proteins. Sph treatment had no effect on mRNA levels of these genes (Figure 2A). In contrast, the protein levels of α-actinin and vinculin that were downregulated in MOSE-L cells compared to MOSE-E cells were modestly but significantly increased after Sph treatment of MOSE-L cells by 21% (from 43 to 62%) and 16% (from 39 to 55%) (*p* < 0.05), respectively; there was no change in these proteins in the Sph-treated MOSE-E (Figure 2B,C). In contrast, prolonged Sph treatment led to a modest decrease in FAK protein levels in MOSE-L cells; however, this was not statistically significant.

**Figure 3:**
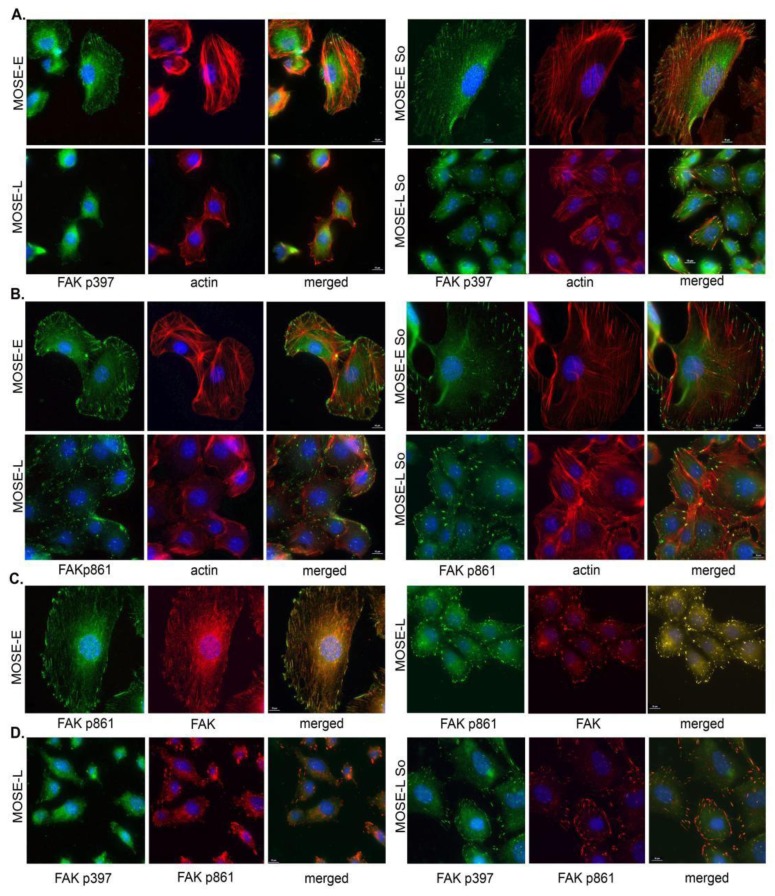
Effects of Sph treatment on FAK phosphorylation. MOSE-E and MOSE-L cells were treated with sphingosine (So) or vehicle, and immunostained for FAK phosphorylated on (**A**) Tyr-397; or (**B**) Tyr-861; (**C**) Co-localization of non-phosphorylated FAK and FAK; (**D**) co-localization of FAK phosphorylated on Tyr-861 and Tyr-397

Since the activation of FAK involves phosphorylation on specific serine and tyrosine residues, we compared the phosphorylation on Tyr-397 (autophosphorylation site), a binding site for Src, and Tyr-861, phosphorylated subsequent to Src binding. As shown in Figure 3A, little phosphorylated p397 FAK could be detected in MOSE-L cells compared the MOSE-E; however, in both cell lines, p397 FAK co-localized to the end of actin bundles. Sph treatment induced FAK phosphorylation on Tyr-397 in more than 50% of the cells, and was strongest when associated with more prominent actin fibers. In contrast, p861 FAK was evident in all MOSE cells (Figure 3B), co-localizing with FAK (Figure 3C) and p397 FAK(Figure 3D). and p397 FAK (Figure 3D). This indicates that while Sph treatment has an only modest effect on FAK protein expression levels, it leads to increases in its Tyr-397 phosphorylation, supporting the establishment of a signaling complex that could promote the formation of actin bundling and FA assembly [35].

### 2.4. Sph Treatment Enhanced Focal Adhesion (FA) Formation and Maturation

**Figure 4:**
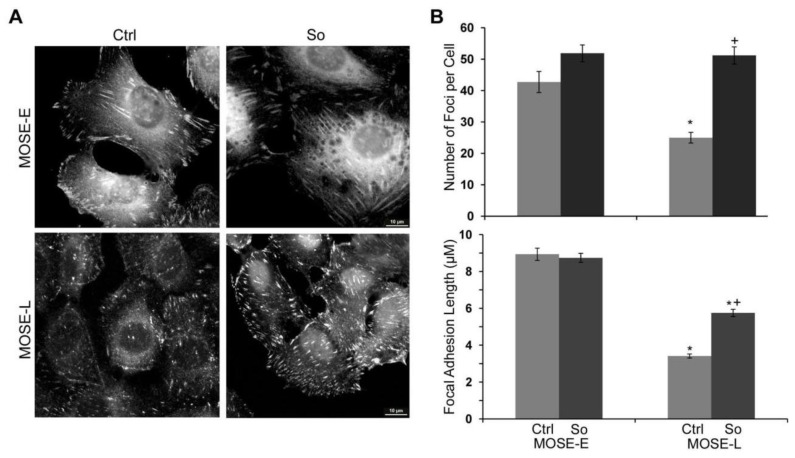
Increased focal adhesion assembly and maturation by treatment with non-toxic concentrations of Sph. MOSE-E and –L cells were treated with 1.5 µM sphingosine (So) or vehicle (Ctrl); FA were visualized by staining for FAK and foci were counted and measured in cells that were completely in the visual field using the NIS Elements software (Nikon). Data represent mean ± SEM. *significantly different from untreated MOSE-E, p < 0.001; ^+^significantly different from corresponding untreated control, p < 0.001.

The dynamics and maturation of FAs and their associated proteins are inherently linked to actin organization and assembly of actin stress fibers [36]. Thus, we determined the number and size of FAs as an indication of their maturation status and as an indirect measurement of Sph-induced changes in actin bundling activity. As shown in Figure 4, there were less FAs in MOSE-L than in MOSE–E cells (25.0 ± 1.7 and 42.73 ± 3.35 per cell, respectively, * p <* 0.001). The number of FAs increased modestly but statistically not significant in MOSE-E following Sph treatment. In contrast, Sph treatment significantly increased the number of FAs in MOSE-L cells to 51.18 ± 2.74 per cell (*p* < 0.001), completely eliminating the observed differences between the MOSE-E and MOSE-L.

The localization and the length of FAs are indicative of their maturation status. In MOSE-E cells, the FAs were distributed throughout the cell (Figure 4A) and were significantly longer than in MOSE-L cells (8.93 ± 0.33 µm *vs.* 3.42 ± 0.11 µm, *p <* 0.001); this was especially evident in the centrally localized FAs.

In contrast, most of the FAs observed in MOSE-L cells were very short and localized towards the cell periphery. Treatment with Sph did not affect the length of FAs in the MOSE-E cells but in MOSE-L cells, a significant increase from 3.41 ± 0.11 µm to 5.75 ± 0.20 µm (*p* < 0.001) was observed upon Sph treatment. Together, our data indicate that Sph impacts the maturation of focal adhesions in malignant MOSE-L cells to a state more closely associated with a non-malignant, less aggressive phenotype, *i.e*., MOSE-E cells.

### 2.5. Regulation of FA and Actin Bundling

**Figure 5:**
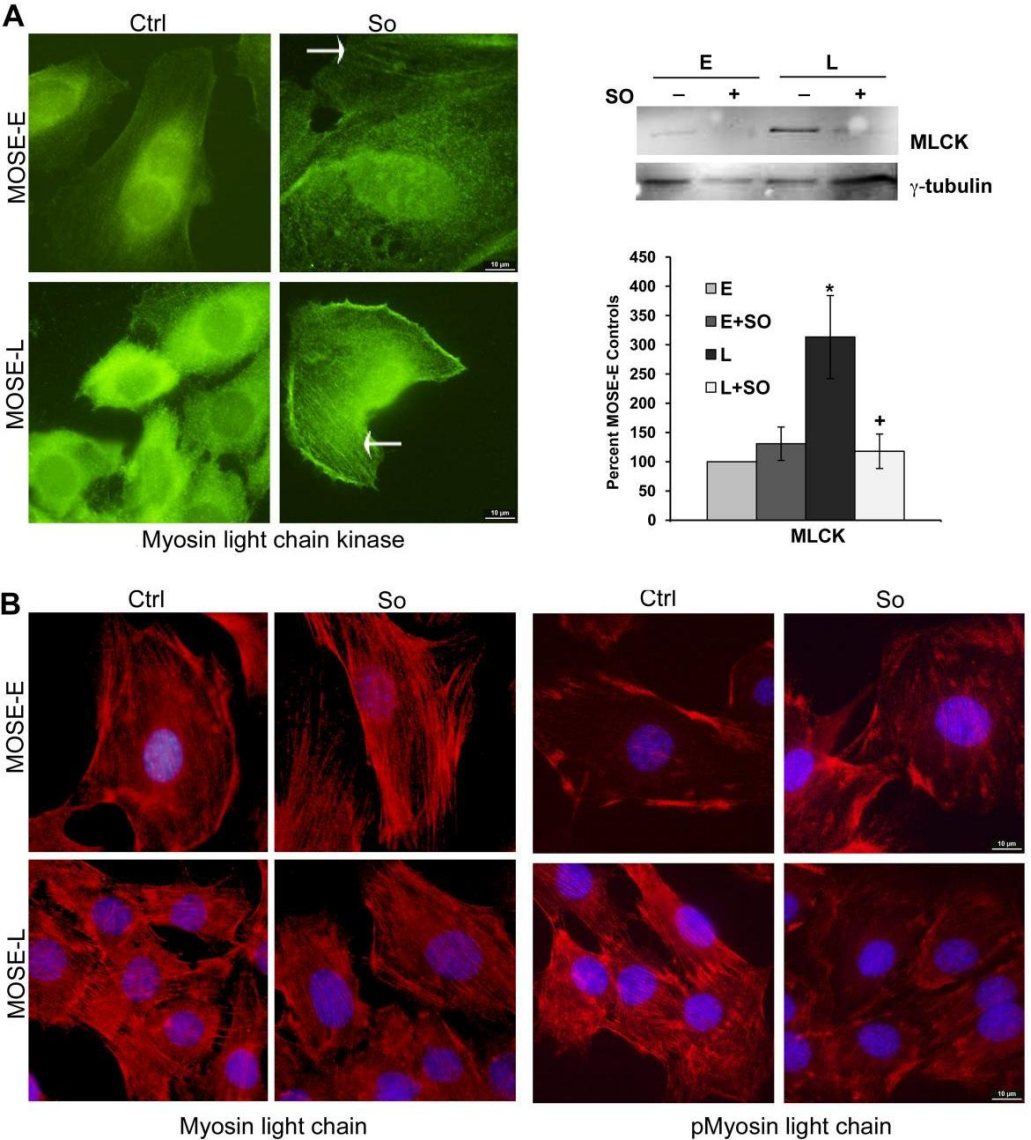
Changes in MLCK expression and its target phosphorylation after Sph treatment. (A) Immunofluorescent identification of myosin light chain kinase (MLCK); arrow indicates co-localization with F-actin (left panel); Western blot analysis and quantitation of MLCK in whole cell extracts, normalized to to γ tubulin. A representative blot is shown in the right panel. Data are expressed as percent of MOSE-E ± SEM; *significantly different from MOSE-E, p < 0.05; +significantly different from corresponding untreated control, p < 0.05. (B) Myosin light chain expression (left panels) or myosin light chain phosphorylated at Ser-19 (pMyosin light chain).

The assembly of structural and signaling proteins that comprise FAs and connect the actin cytoskeleton to the extracellular matrix is regulated by several post-translational modifications that modulate both assembly and the physiological response. One downstream target of FA signaling is myosin light chain kinase (MLCK), a Ca^2+^ and calmodulin-dependent kinase that phosphorylates MLC on T18 and S19. This increases myosin II filament formation, actin-myosin interactions, and myosin ATP activity that facilitates myosin-driven actin filament movement critical for migration and invasion of non-muscle cells [37], and identifies MLCK as a key player in cancer metastasis. We found a significant increase of MLCK protein levels in MOSE-L cells (3.5 fold, *p <* 0.001) that was completely abrogated by Sph (Figure 5A,B). Furthermore, there was a higher incidence of co-localization of MLCK with actin fibers in Sph treated cells which corresponds to the observed effects of Sph on the organization of the actin cytoskeleton (Figure 5A, right panels, arrows). We next determined the phosphorylation status of the MLC, the target of MLCK activity. The levels of MLC were elevated in MOSE-L cells with little to no change by Sph treatment Figure 5B). In contrast, MLC phosphorylation on S19 was markedly increased in the MOSE-L cells. Importantly, MLC phosphorylation on S19 was almost completely abrogated by Sph (Figure 5B, right panels). It is currently unknown whether Sph in non-toxic concentrations directly inhibits the activity of MLCK as has been shown for concentrations >50 µM Sph in purified enzyme assays [38].

### 2.6. Suppression of Invasion *in vitro*

**Figure 6:**
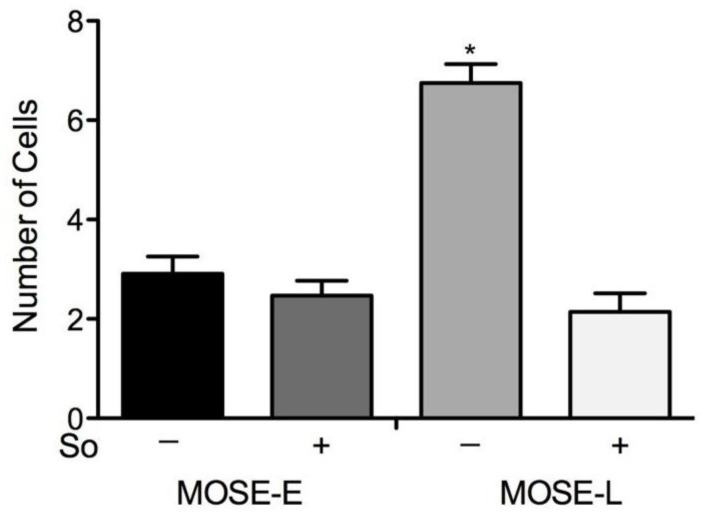
Suppression of invasion by Sph treatment. Number of cells able to invade matrigel afte r sphingosine (So) treatment. Date are presented as mean ± SEM. * significantly different from MOSE-E, *p* < 0.01.

Changes in the cytoskeleton can affect cellular functions such as invasion. To determine the invasion potential of the MOSE cells, cells able to invade matrigel-coated filter inserts towards a chemoattractant were counted. As shown in Figure 6, the invasive capacity of MOSE-L cells was significantly greater than that of MOSE-E cells (*p* < 0.01). However, treatment with Sph significantly reduced invasion in MOSE-L cells (*p* < 0.01) but did not affect MOSE-E cells.

### 2.7. Discussion

Metastatic tumor cells require active modulation of their cytoskeleton architecture to adapt to changing microenvironments. We previously reported the prominent, step-wise dysregulation of the cytoskeleton during the progression of the pre-malignant MOSE-E cells to malignant and metastatic MOSE-L cells. In the present study, we investigated whether treatment with concentrations of exogenous Sph at levels that reduce the tumorigenic and metastatic potential of the MOSE cells but do not induce apoptosis (Schmelz *et al.,* unpublished observations) affect the actin cytoskeleton and its regulation in the MOSE cells. We found that Sph treatment increased polymerized F-actin levels in malignant MOSE-L cells and increased actin cytoskeleton organization. Coinciding with an increase in microfilament bundling, Sph treatment also increased vinculin and α-actinin expression levels, which were decreased in malignant MOSE-L cells compared to their benign precursor cells. Furthermore, Sph increased the number and the size of FAs and increased FAK phosphorylation on Tyr-397, suggesting that Sph promotes both FA maturation and increases actin bundling activity. This was associated with the downregulation of the high levels of MLCK in aggressive MOSE-L cells, and ultimately led to a reduced rate of phosphorylation of the MLCK target, MLC. Together, our results indicate that the cellular cytoskeleton regulatory machinery is a target for Sph, and the observed partial or complete reversal of progression-associated changes may contribute to an anti-cancer effect of So as indicated by the suppression of invasion *in vitro*.

Sphingolipid metabolites have been shown to modulate the cytoskeleton and its regulatory proteins in other tissues. Cer has been shown to induce stress fibers in fibroblasts [39]; in breast cancer cells, Cer is involved in cisplatin-induced dissociation of actin filaments from the membrane [30] while in colon cancer cells, Cer altered several cytoskeleton-associated proteins including tubulin ß-5, ß-actin, and tropomyosin β-3 [40].S1P also plays a prominent role in cytoskeleton reorganization and has shown to rearrange the microfilament network by modulating FAK, α-actinin, and paxillin via Rac1 and Rho GTPase activity in endothelial cells and fibroblasts [41,42]. The induction of stress fibers via S1P receptor signaling has been reported in many cell lines and dependent on the cell type, induce changes in cell motility, angiogenesis, myocardial development, tumor cell dissemination, immune cell differentiation and migration, contractility, endothelial barrier function, neurite outgrowth and more [29,43]. This is especially relevant in ovarian cancer since ascites fluid is enriched in epithelial ovarian cancer patients; concentrations between 0.04 to 2 µM have been reported [44,45]. The increased migration and invasion observed in ovarian cancer cells in response to physiologically relevant S1P concentrations [44] suggest that S1P contributes to tumor cell survival, growth and metastasis. Both S1P and Cer modulate the phosphorylation of the critical actin-regulating proteins ezrin, radixin, and moesin, with S1P increasing and Cer decreasing phosphorylation [46].

Sph has been shown to induce actin stress fibers and FA assembly by promoting the phosphorylation of FAK in fibroblasts [47]. Sph also inhibits protein kinase C [48] and myosin light chain kinase [38], major regulators of the actin cytoskeleton. Thus, sphingolipid metabolites are critically involved in the regulation of the cytoskeleton. Our studies show a significant increase in F-actin polymerization and cytoskeleton organization after Sph treatment. This was not mediated by the conversion of Sph to S1P by SK1, an enzyme that is up-regulated in MOSE-L cells (unpublished observations), since enigmol, a synthetic Sph analog lacking the C1-hydroxyl group and therefore is not a substrate for SK1, induced similar actin reorganization and increased F-actin levels. Furthermore, S1P treatment only marginally induced stress fibers in a small sub-population of cells after short-term but not longer treatment. Enigmol can also be acylated to Cer albeit at a slow rate [49]. Cer has been shown to cause a disruption of the actin cytoskeleton in cancer cells [30] and decreases F-actin [31]. Our results also show that Cer alters actin organization in MOSE-E cells but rather than a strengthening of actin cables, we observed the opposite effect. While the conversion of Sph to other metabolites cannot be excluded at this time, our results suggest that Sph is at least partially responsible for the observed changes in the cytoskeleton organization. If the observed changes by DHCer and S1P treatment affect ovarian cancer progression or tumorigenic potential need to be examined in more detail.

An increase in F-actin or its stabilization has been shown to be associated with higher cell rigidity [50] and provides resistance against deformability [51]. Likewise, a decrease in α-actinin (also observed in our study) reduces actin crosslinking and actin fiber anchoring in FAs [52]. Our studies have shown that changes in the actin but not tubulin cytoskeleton reduced cell viscoelasticity during ovarian cancer progression [11,12]; furthermore, changes in the cytoskeleton and membrane organization impact the cells’ bioelectrical properties [13,14]. Of note, Sph but not S1P decreased membrane capacitance (the cells ability to store electric charge) and the membrane-specific area parameter φ, a measure for surface roughness (determined by protrusions, ruffles, folds, microvilli, blebs *etc.*) in MOSE-L cells; the latter was increased by S1P [53], indicating that MOSE-L cells exhibit a smoother surface after Sph treatment but a rougher surface after treatment with S1P. These events are of particular interest because changes in the biomechanic and biolelectrical properties of the cancer cells may affect their metastatic potential.

The number of FAs and their size is an indication of their maturation status. FAs connect the actin cytoskeleton to the membrane and the extracellular matrix, thereby mediating extracellular signals to the inside of the cell. Their dynamic assembly and turnover is required for proliferation and the regulation of adhesion and motility. Stabilized FAs or reduced FA turnover associated with increased actin stress fiber formation and FA maturation increases adhesion and inhibits motility [54,55]. α-actinin and vinculin are important structural components of FAs serving to link actin to integrins, and mediate protein-protein interactions involved in forming and maintaining FAs [56]. The loss of vinculin has been shown to increase FA turnover and thereby increase cell spreading and motility [55]. If Sph–induced increases in α-actinin and vinculin protein levels are linked to the observed increase in FA numbers and maturation status needs further investigation. Interestingly, Tyr-397 phosphorylation which activates FAK has been shown to increase actin bundling [35] but has also been shown to be elevated in ovarian cancer cells in response to stress, and in ovarian tumors [57]. FAK functions as kinase and scaffold, modulating processes such as actin cytoskeleton organization, motility, adhesion, proliferation, gene expression and more in a context-dependent manner (see review [58,59]); the mechanism(s) of how Sph increases Tyr-397 phosphorylation and differentially impacts these functions this needs to be investigated in more detail.

Downstream targets of FA signaling include MLCK, one of the major regulators of the actin myosin network. MLCK was elevated in the MOSE-L cells, consistent with observations in other metastatic cell lines [60]. MLCK increases proliferation and migration of breast cancer cells [61]. Via activation by ERK, MLCK promotes FA turnover and phosphorylation of MLC [56] which is required for actin-myosin mediated motility [37]. MLC phosphorylation is essential for the switch from a dormant to proliferative phenotype of breast cancer cells grown in 3D culture [3]. Interestingly, MLC hyperphosphorylation has been associated with the induction of actin stress fibers in breast cancer and endothelial cells [3]. Here we report that Sph treatment leads to a hypophosphorylated MLC and this coincided with enhanced actin organization and stress fiber formation. The inhibition of MLCK and, subsequently, MLC phosphorylation alters cell polarization and motility [61], induces apoptosis in cancer cells both *in vivo* and *in vitro*, and suppresses tumor formation [62], indicating an inherent link between FAs, MLCK and MLC phosphorylation in cancer growth. Whether Sph-mediated changes on MLCK expression and activity are direct effects or mediated via modulation of other signaling intermediates remains to be determined.

## 3. Experimental Section

### 3.1. Cell Culture

The MOSE cells utilized in the present studies were classified into early (MOSE-E, passages 14–18) and late (MOSE-L, passages 175–185) stages based on their morphology, growth characteristics and tumorigenic potential [8]. MOSE cells were routinely maintained in DMEM high glucose medium (Invitrogen) supplemented with 4% fetal bovine serum (Hyclone), and 100 mg/mL each of penicillin and streptomycin. Parallel passages were grown in vented tissue culture flasks in the presence or absence of 1.5 µM Sph (Avanti Polar Lipids) as BSA complex (fatty acid-free fraction V, Calbiochem), for the indicated times or a minimum of three passages mimicking long-term exposure to diet-derived sphingolipid metabolites. The sphingoid bases are the likely metabolites involved in the observed suppression of tumor development and progression [63] and therefore used in this study. In parallel, cells were incubated with 2.0 µM C8Cer, 2.0 µM C8-dihydroceramide (DHCer), 500 nM S1P (Avanti), or 1.0 µM enigmol (from Dr. A.H. Merrill, Georgia Tech, Atlanta, GA) for 3 passages, seeded into 100 mm tissue culture dishes, and grown to 60–70% confluency.

### 3.2. Immunofluorescent Staining

Cells grown on glass cover slips to 50-60% confluency were fixed and immunostained as described [8,9]. Monoclonal antibodies recognizing vinculin (Sigma), focal adhesion kinase (FAK) (Millipore), phosphorylated FAK on Tyr-397 (ECM Bioscience) or Tyr-861 (Sigma), α-actinin (Abcam), myosin light chain kinase (MLCK) (Sigma), myosin light chain (MLC) 2, or myosin light chain 2 phospho-serine 19 (Cell Signaling) were used in paraformaldehyde-fixed cells. Methanol fixation was used for tubulin (Sigma) or cytokeratin (Sigma) immunostaining. Secondary antibodies or phalloidin conjugated to AlexaFluor^594^ or AlexaFluor^488^ were from Molecular probes. Incubations with secondary antibodies alone were performed to determine the background staining. Coverslips were mounted onto glass slides using Prolong Gold antifade reagent with DAPI (Invitrogen).Immunofluorescence was observed using a 60X objective on a Nikon 80*i* epifluorescence microscope equipped with UV, FITC and TRITC filters, DS-Fi1 color and DS-U2 monochromatic cameras. Images were captured and analyzed using NIS Elements BR 3.0 software (Nikon Instruments, Inc.) and processed using Adobe Photoshop^®^. To compare protein expression levels and localization, care was taken to ensure that pictures were taken with the same exposure time.

### 3.3. Quantitation of Filamentous Actin

Cells were seeded at 2000 cells per well in a 96 well plate and treated with vehicle or 1.5 µM Sph for the times indicated, or passaged 3-5 time in 1.5 µM Sph before plating. After 48h, cells were fixed in 3% PFA for 10 min, permeabilized in 6% PFA with 0.5% Triton X-100 for 10 min, quenched with 50 mM glycine, followed by a 60 min blocking step with 2% chicken serum. F-actin was stained with AlexaFluor488- conjugated phalloidin for 30 min, followed by extensive washings to remove unbound phalloidin.

Phalloidin was solubilized with MeOH, transferred to an assay plate, and the fluorescence was determined with an exitation wavelength of 488 nm and emission of 525 nm using a Safire2 microplate reader (Tecan) with Magellan v6.3 for windows software. A parallel set of cells was used to determine cell number using the MTT assay as described previously [64] or the Cyquant Cell Proliferation Kit (Invitrogen) according to the manufacture’s parameters. Data from three independent experiments are expressed as mean relative fluorescence per cell ± SEM.To quantitate F-actin in cells treated with Cer, DHCer, enigmol, and S1P, cells were stained with hematoxylin after methanol extraction and 3 pre-determined fields per well were counted for normalization.

### 3.4. Western Blot Analysis

Cells were grown in the presence or absence of 1.5 µM Sph for 48 h to 50% confluency in 100mm dishes, harvested by scraping into RIPA buffer, and proteins were separated by SDS-PAGE and Western blotting as described using monoclonal antibodies directed against vinculin, β-tubulin, and β-actin (Sigma), FAK (Millipore) and α-actinin (Abcam). Protein bands were visualized on the Chemidoc (Bio-Rad). Densitometric quantitation of relative band intensity was performed using the NIH Image J program and normalized to γ-tubulin. Data is expressed as percent of controls and is the mean of 3–4 biological replicates done in duplicate.

### 3.5. Quantitative Real-Time Polymerase Chain Reaction PCR (qRT-PCR)

Total RNA was extracted from biological replicate samples grown as described above. 500 ng of total RNA was reverse-transcribed using the ImProm-II Reverse Transcription System (Promega) with random hexamer and oligo-dT primers according to the manufacturer’s instructions. Quantitative Real-time Polymerase Chain Reaction (qRT-PCR) was performed on 5 ng of cDNA using gene specific primers designed using Beacon Design software and SensiMix Plus Sybr mastermix (Quantace) in 15 µL reaction volume. qRT-PCR was performed for 42 cycles at 95 °C for 15 s, 56–58 °C for 30 s, and 72 °C for 15 s, preceded by a 10 min incubation at 95 °C, on the ABI 7900HT Fast Real-Time PCR System (Applied Biosystems). Melt curves were performed to insure fidelity of the PCR product. All primer pairs displayed efficiency between 90-105% and sample amplification was within the dynamic range of the primers. L19 was used as housekeeping gene and the ΔΔCt method [65] was used to determine fold difference; the student t-test was utilized to ascertain significance. Data represent the mean ± SD of three biological replicates done in duplicates.

### 3.6. Cell Invasion Assay

MOSE-E and -L cells were serum starved overnight, and seeded in serum-free DMEM into the upper chamber of migration/invasion chambers (Becton Dickenson) coated with matrigel, 8 µM pore size). The lower chamber contained DMEM with 5% FCS. The cells were incubated for 6h to avoid an impact of the different doubling times of the cell lines [8]. The cells remaining in the upper chamber were wiped off with a cotton swap and cells that had migrated to the other side of the membranes were fixed and stained with the Diff-Quick staining kit as recommended by the manufacturer (Andwin Scientific). The membranes were removed, mounted onto glass slides and five areas per slide in a pre-defined pattern on a grid were counted at 10X magnification. Data are expressed as mean ± SEM of 2 independent experiment performed in triplicate or quadruplicate.

### 3.7. Statistics

Data are expressed as mean ± SEM or SD from at least two independent experiments performed in duplicate or triplicate. Differences between groups were calculated with the Tukey test performed after ANOVA indicated significant differences. Instat® (GraphPad Software) or EZ ANOVA software were used for the statistical analyses.

## 4. Conclusions

Exogenous Sph treatment led to a reversal of a cytoskeleton architecture associated with aggressive cancer phenotypes towards a more stabilized organization reminiscent of benign cell phenotypes. The reversal resulted in increased F-actin polymerization and organization, a changed FAK phosphorylation, increased α-actinin and vinculin levels and led to the assembly of more mature FAs. Concomitantly, a suppression of MLCK expression and hypophosphorylation of MLC were observed. This was associated with a reduced invasive capacity of the Sph-treated cells. If these events are leading to the suppression of ovarian cancer growth, progression and perhaps metastatic outgrowth and could be used as a biological marker for sphingolipid efficacy *in vivo* warrants further investigation.

## Supplementary Files

Supplementary File 1 Supplementary Picture (JPG, 755 KB)
