# Supplementary figures and images for: Regulation of Cytoskeleton Organization by Sphingosine in a Mouse Cell Model of Progressive Ovarian Cancer
Source: Biomolecules. 2013 Jul 16;3(3):386–407. doi: 10.3390/biom3030386 (PMC4030958; doi:10.3390/biom3030386)

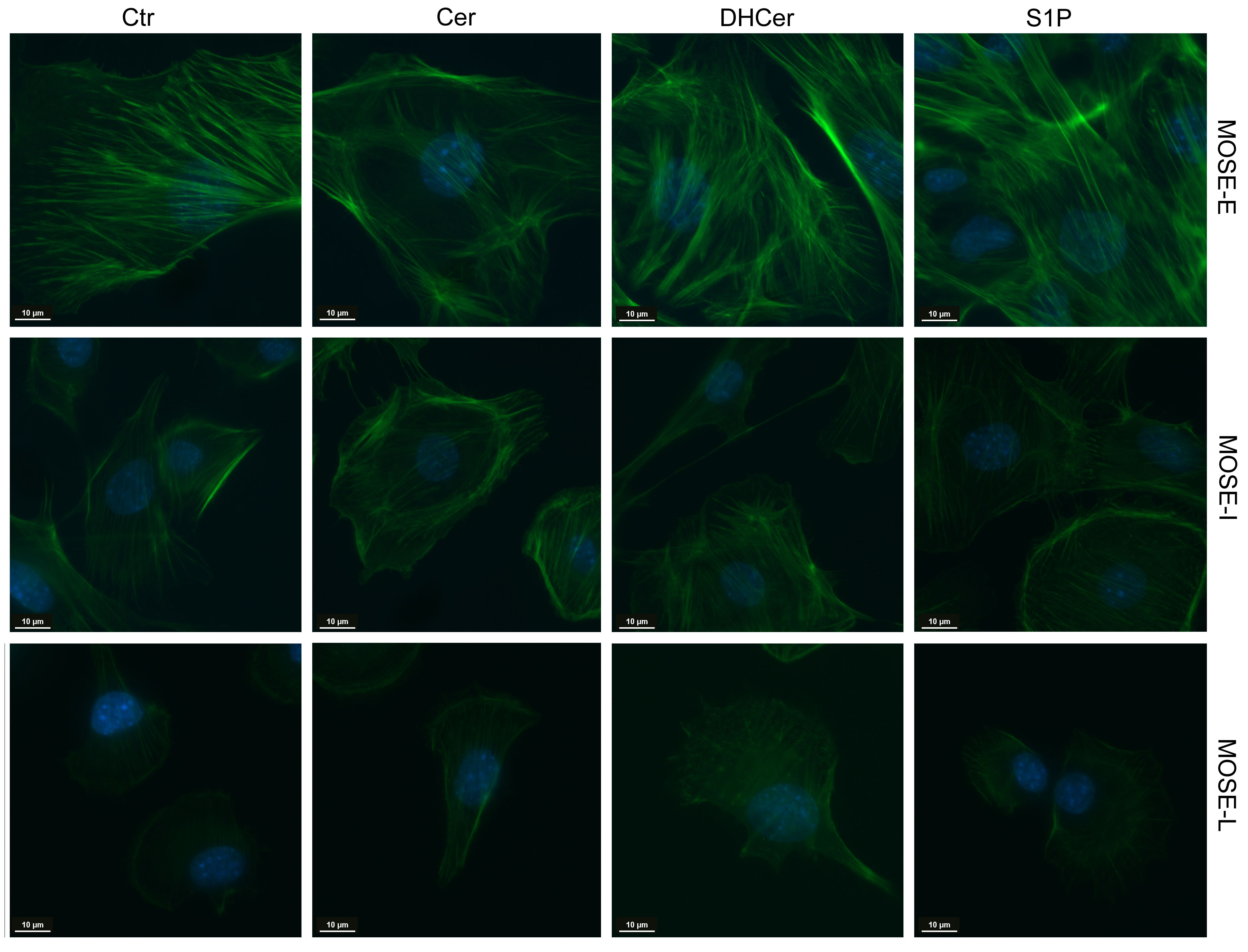

Supplement: Supplementary File 1 — Supplementary Picture (JPG, 755 KB) [file biomolecules-03-00386-s001.jpg]
